# Complete genome sequence of *Trueperella bernardiae* strain UMB8254, isolated from the bladder of a female with metabolic syndrome and nephrolithiasis

**DOI:** 10.1128/mra.01265-24

**Published:** 2025-03-21

**Authors:** Robert B. Moreland, Brian I. Choi, Melline Fontes Noronha, Josh Baker, Jacob Kaindl, Alan J. Wolfe

**Affiliations:** 1 Department of Microbiology and Immunology, Stritch School of Medicine, Loyola University Chicago, Maywood, Illinois, USA; University of Maryland School of Medicine, Baltimore, Maryland, USA

**Keywords:** *Trueperella bernardiae*, metabolic syndrome, nephrolithiasis, kidney stone, Urobiome, human microbiome, genomics

## Abstract

*Trueperella bernardiae* is infrequently isolated, usually in polymicrobial communities, from human hosts with a wide variety of symptoms and diseases. Here, we report a complete genome sequence of *Trueperella bernardiae* (UMB8254), isolated from the bladder of a human female with metabolic syndrome and nephrolithiasis.

## ANNOUNCEMENT

*Trueperella bernardiae* is a Gram-positive, pleomorphic, non-spore-forming, non-motile, non-encapsulated, facultative anaerobic rod that is characterized by fermentative metabolism and strong proteolytic activity. This catalase-negative coccobacillus is an opportunistic and emerging pathogen in both humans (1–10) and animals (11, 12). Initial isolates recovered from blood, bone, abscess, and skin infections were first described using the provisional name CDC fermentative Coryneform group 2 (CDC group 2) (12). After phylogenetic reassessment, this species was reassigned to *Trueperella* (13). Within this genus, only *T. bernardiae* and *T. pyrogenes* have been reported in humans, all associated with mild to severe infections and abscesses such as sepsis following pyelonephritis (14). It has yet to be established if *T. bernardiae* is commensal or opportunistically pathogenic within skin, oropharynx, or urinary tract microbiomes (10, 11, 13). Before the advent of matrix-assisted desorption ionization-time of flight mass spectrometry (MALDI-TOF MS), accurate identification of bacterial species such as *T. bernardiae* in routine laboratories was difficult (14). *T. bernardiae* tends to be found in polymicrobial infections, and immunosuppressed patients appear to be more at risk for infection (15).

In this study, we present the complete genome sequence of *T. bernardiae* strain UMB8254. This strain was isolated on 31 January 2018 from bladder urine obtained via transurethral catheter from a female urology patient at Loyola University Medical Center, Maywood, Illinois, United States, as part of a prospective study observing microbes associated with kidney stone promotion, following Loyola University’s institutional review board approval (LU208983) (16). This patient had been diagnosed with nephrolithiasis and was identified as meeting metabolic syndrome criteria according to the National Cholesterol Education Program Adult Treatment Panel III definition (17). The strain was originally identified as *T. bernardiae* via MALDI-TOF mass spectrometry, as previously described (18). This method involved incubating urine on sheep blood agar plates for 48 hours at 37°C with 5% supplemented CO_2_ gas followed by single-colony isolation, mass spectrometry analysis, and cryopreservation. For isolation of DNA, a sample was cultured from cryopreservation using the same conditions and method as identification.

DNA extraction and sequencing for long-read Oxford Nanopore sequences of this strain was performed by SeqCenter using ZymoBIOMICS DNA Miniprep Kit and Oxford Nanopore Technology Ligation Sequencing kit V14 with the NEBNext Companion Module according to the manufacturer’s specifications. No additional DNA fragmentation or size selection was performed. Sequencing was conducted on R10.4.1 flowcells run on the MinION platform. Guppy v6.4.6 (Nanopore) was used for basecalling, demultiplexing, and adapter removal.

Assembly was performed using Oxford Nanopore sequence reads. Default parameters were used for all software unless otherwise specified. NanoFilt v2.8.0 (19) was used to filter low-quality sequences with quality assessment conducted by fastQC v0.11.9 (20) and NanoStat v1.6.0 (19). High-quality reads were used for the assembly using Flye v.2.9 (21). The genome assembly qualities were validated with QUAST v5.2.0 (22) and circularized using circlator v1.5.5 (23). PGAP v6.7 (24) was used for genome annotations (Table 1). Filtering for “strict hits” via the Comprehensive Antibiotic Resistance Database (CARD) (25) yielded a hit to the gene, *ErmX*, indicating potential macrolide resistance.

**TABLE 1:** Complete genome characteristics

Characteristic	UMB8254
Nanopore sequencing	
No. of reads	328,651
Read *N*_50_ (bp)	5,485
Genome properties	
No. of contigs	1
Structure	Circular
Total genome length (bp)	2,103,060 bp
GC content (%)	65.19%
Coverage (×)	489×
Predicted no. of coding DNA sequences	1,834
No. of rRNAs (5S, 16S, 23S)	2, 2, 2
No. of tRNAs	46
GenBank accession no.	CP127099

## Data Availability

The Nanopore raw reads/assembly has been deposited in GenBank under the following accession numbers: SRR25068138/CP127099
